# Habit Formation after Random Interval Training Is Associated with Increased Adenosine A_2A_ Receptor and Dopamine D_2_ Receptor Heterodimers in the Striatum

**DOI:** 10.3389/fnmol.2016.00151

**Published:** 2016-12-26

**Authors:** Yan He, Yan Li, Mozi Chen, Zhilan Pu, Feiyang Zhang, Long Chen, Yang Ruan, Xinran Pan, Chaoxiang He, Xingjun Chen, Zhihui Li, Jiang-Fan Chen

**Affiliations:** ^1^School of Optometry and Ophthalmology and Eye Hospital, Institute of Molecular Medicine, Wenzhou Medical UniversityWenzhou, China; ^2^Department of Geriatrics and Neurology, The 2nd Affiliated Hospital & Yuying Children's Hospital of Wenzhou Medical UniversityWenzhou, China; ^3^Ma'anshan Municipal Hospital Group and Municipal People's HospitalMa'anshan, China; ^4^Department of Neurology, School of Medicine, Boston UniversityBoston, MA, USA

**Keywords:** A_2A_ receptor, D_2_ receptor, receptor-receptor heterodimers, goal-directed behavior, habit, striatum

## Abstract

Striatal adenosine A_2A_ receptors (A_2A_Rs) modulate striatal synaptic plasticity and instrumental learning, possibly by functional interaction with the dopamine D_2_ receptors (D_2_Rs) and metabotropic glutamate receptors 5 (mGluR5) through receptor-receptor heterodimers, but *in vivo* evidence for these interactions is lacking. Using *in situ* proximity ligation assay (PLA), we studied the subregional distribution of the A_2A_R-D_2_R and A_2A_R-mGluR5 heterodimer complexes in the striatum and their adaptive changes over the random interval and random ratio training of instrumental learning. After confirming the specificity of the PLA detection of the A_2A_R-D_2_R heterodimers with the A_2A_R knockout and D_2_R knockout mice, we detected a heterogeneous distribution of the A_2A_R-D_2_R heterodimer complexes in the striatum, being more abundant in the dorsolateral than the dorsomedial striatum. Importantly, habit formation after the random interval training was associated with the increased formation of the A_2A_R-D_2_R heterodimer complexes, with prominant increase in the dorsomedial striatum. Conversely, goal-directed behavior after the random ratio schedule was not associated with the adaptive change in the A_2A_R-D_2_R heterodimer complexes. In contrast to the A_2A_R-D_2_R heterodimers, the A_2A_R-mGluR5 heterodimers showed neither subregional variation in the striatum nor adaptive changes over either the random ratio (RR) or random interval (RI) training of instrumental learning. These findings suggest that development of habit formation is associated with increased formation of the A_2A_R-D_2_R heterodimer protein complexes which may lead to reduced dependence on D_2_R signaling in the striatum.

## Introduction

The adenosine A_2A_ receptors (A_2A_Rs) are highly enriched in the striatopallidal neurons of the striatum (Svenningsson et al., 1999) where A_2A_Rs are co-localized with and form heterodimers with the dopamine D_2_ receptors (D_2_Rs) and metabotropic glutamate 5 receptors (mGluR5) (Tebano et al., 2009; Pinna et al., 2014; Taura et al., 2015). Possibly through the receptor-receptor heterodimerization, striatopallidal A_2A_Rs interact antagonistically with D_2_Rs (Canals et al., 2003; Trifilieff et al., 2011), and synergistically with mGluR5 (Ferré et al., 2002; Kachroo et al., 2005). By these functional interactions, striatopallidal A_2A_Rs can modulate dopamine and glutamate signaling and striatal synaptic plasticity and cognitions including instrumental behaviors (Chen, 2014). Indeed, genetic inactivation of striatal A_2A_Rs impaired habit formation (Yu et al., 2009) and pharmacological reduction of A_2A_R-mediated cAMP-pCREB signaling in the dorsal medium striatum (DMS) enhanced goal-directed ethanol drinking (Nam et al., 2013) and reversed meth-amphetamine-induced facilitation of habit formation (Furlong et al., 2015). However, the mechanism underlying the A_2A_R modulation of instrumental behaviors is not known.

Striatal long-term depression (LTD) that is restricted to striatopallidal neurons and requires activation of D_2_Rs and mGluR5 (Kreitzer and Malenka, 2007; Lovinger, 2010) is the main form of plasticity of synaptic transmission in the dorsolateral striatum (DLS; Partridge et al., 2000; Yin and Knowlton, 2006; Lovinger, 2010). The loss of striatopallidal LTD is associated with a shift in behavioral control from goal-directed (Furlong et al., 2015) action to habitual responding (Nazzaro et al., 2012). Since activation of striatopallidal A_2A_Rs can convert the striatopallidal synaptic plasticity from LTD to long-term potentiation (LTP; Shen et al., 2008), striatopallidal A_2A_R signaling may interact with D_2_R-/mGluR5-/endocannabinoids-mediated striatal LTD in striatopallidal neurons to modify instrumental learning. Thus, we postulated that striatopallidal A_2A_Rs may exert their effects on D_2_R- or mGluR5-mediated striatal synaptic plasticity and instrumental learning through the physical association of the A_2A_R-D_2_R and A_2A_R-mGluR5 heterodimers in the striatopallidal neurons. Here, using two instrumental learning schedules coupled with *in situ* proximity ligation assay (PLA), we investigated the heterogeneous distribution of the A_2A_R-D_2_R and A_2A_R-mGluR5 heterodimers in the DLS and DMS and their adaptive changes after the random interval (to promote habit) and random ratio (to promote goal-directed behavior) training schedules.

## Materials and methods

### Animals

All animals were handled in accordance with the protocols approved by the Institutional Ethics Committee for Animal Use in Research and Education at Wenzhou Medical University, China. Eighteen adult C57B6/J (*n* = 6/experimental group), three A_2A_R knockout mice (from Chen's laboratory at Boston University School of Medicine) and three D_2_R knockout mice (from The Jackson Laboratory, USA, Drd2^tm1Low^, stock No. 003190) were used for the experiments.

### Instrumental behavior training schedules

Instrumental training and behavioral testing schedules were performed following the procedure by Rossi et al. (Rossi and Yin, 2012). In brief, mice first underwent a 5-day food deprivation schedule to reach 80–85% of their free-feeding weight before instrumental training sessions. Mice were then given one 30-min magazine training session during which one drop of 20 μl 20% sucrose solution as reward was delivered on a random time 60-s schedule. During continuous reinforcement (CRF) sessions, each lever press resulted in delivery of the sucrose reward. Sessions ended after 60 min or 50 rewards had been earned, whichever came first. After CRF, mice underwent a random interval (RI) schedule to promote habit formation or a random ratio (RR) schedule to promote goal-directed behavior. Mice underwent the RI schedule were trained for 2 days on random interval 30 s (RI30), with a 0.1 probability of reward availability every 3 s contingent upon lever pressing, followed by 4 days on RI60 schedule. Progressively leaner schedules of reinforcement were used for the RR training procedure: RR5 (each response was rewarded at a probability of 0.2 on average), RR10, RR20 each for 2 days.

Following the RI and RR training sessions, a 2-day devaluation test was conducted. A specific satiety procedure was applied to alter the current value of a specific reward. On each day the mice were allowed to have free access to home chows which were used for maintaining their weights (i.e., valued condition, the sucrose solution was still valued) or sucrose solution which was earned by their lever pressing in the training sessions (i.e., devalued condition, the sucrose solution was devalued) for at least an hour to achieve sensory-specific satiety. Immediately after the unlimited pre-feeding session, mice were given a 5-min extinction test during which the lever was inserted and pressing times was recorded but without reward delivery. The orders of the valued and devalued condition tests (day 1 or 2) were counterbalanced across each group. Mice insensitive to manipulation of outcome value, that is habit, would mildly change lever presses on the devalued condition compared to the valued condition, whereas goal-directed mice that performed sensitively to outcome value would significantly reduce their lever presses on the devalued condition. The control mice underwent food deprivation schedule and were handled exactly the same way every day just as the RR and RI training group before instrumental training. During the training sessions, the control mice were also placed in the operant chambers in which the sucrose reward was delivered in a random 30/60 s (corresponding to RI30/RI60) manner but without lever stretched. In the devaluation test, the control mice were also exposed to the operant chamber for 5 min with no lever stretched out and no reward available. In the present study, three groups (*n* = 6 for each group) were examined for the A_2A_R-D_2_R/A_2A_R-mGLuR5 heterodimers in the striatum after instrumental learning: (a) mice without instrumental training as “control group,” (b) mice underwent RI/RR training sessions as “RI group,” and (c) mice underwent RR training sessions as “RR group.”

### Proximity-ligation assay (PLA)

**Figure 1:**
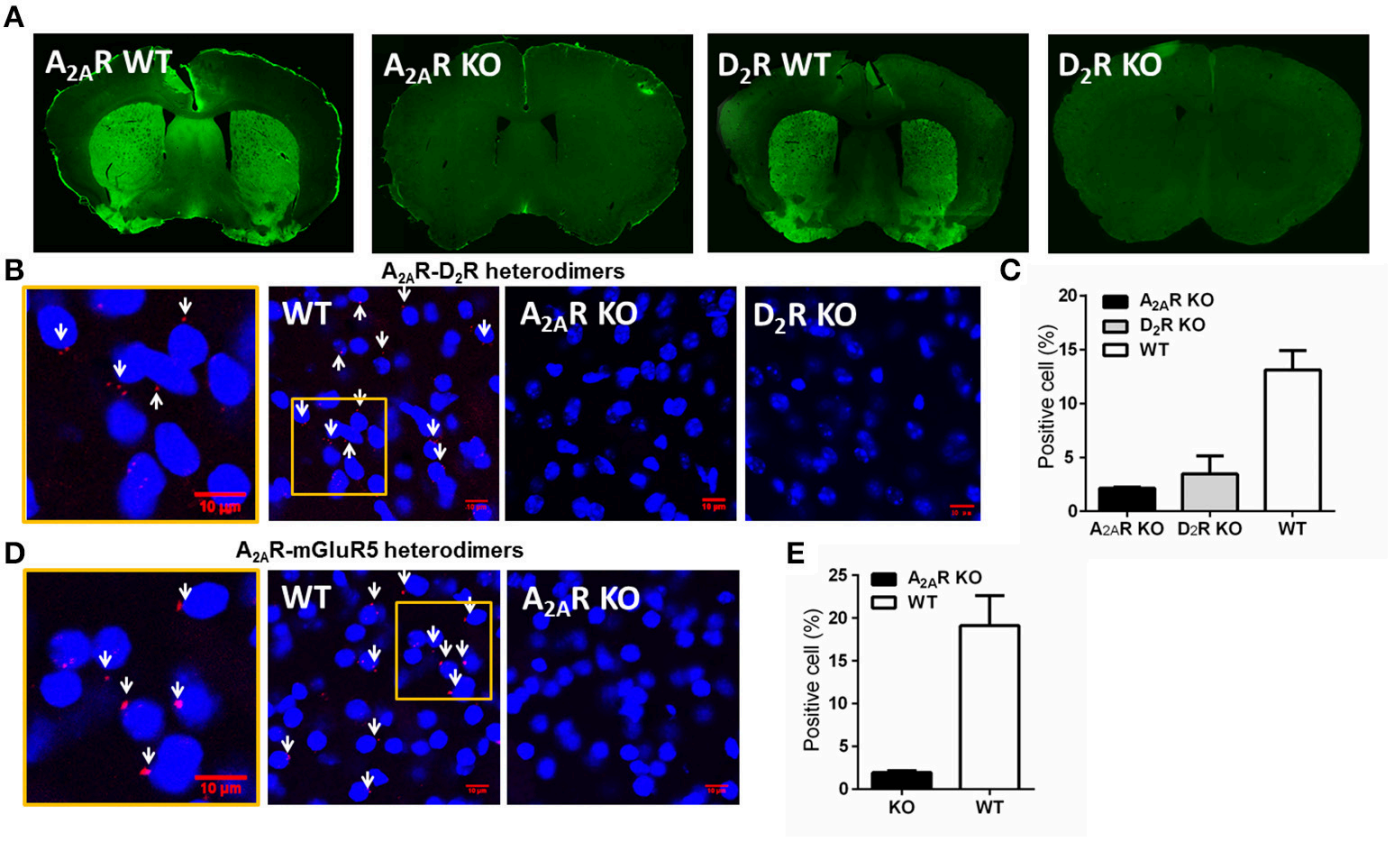
**Detection and specificity of the A_**2A**_R-D_**2**_R and A_**2A**_R-mGluR5 heterodimers in the striatum by PLA. (A)** Representative immunofluorescent photomicrographs show A_2A_Rs and D_2_R were highly expressed in the striatum of WT mice, but were absent in A_2A_R or D_2_R KO mice. **(B)** Physical association of A_2A_R-D_2_R heterodimers was detected by PLA in sections of the striatum from WT mice, but not from A_2A_R KO mice and D_2_R KO mice. The enlarged confocal image (Left) identified the PLA signals (red puncta) and positive cells (white arrows). **(C)** Quantification of the A_2A_R-D_2_R heterodimers by PLA in the striatum of WT (*n* = 6), A_2A_R KO (*n* = 3), and D_2_R KO (*n* = 3) mice. **(D)** PLA signals of A_2A_R-mGluR5 heterodimers were detected in the striatum of WT mice (middle), with amplified image (left), but were absent in A_2A_R KO mice (right). **(E)** Quantification of A_2A_R-mGluR5 heterodimers by PLA for WT (*n* = 6) and A_2A_R KO (*n* = 3) mice. Data are presented as the mean ± SEM.

**Figure 2:**
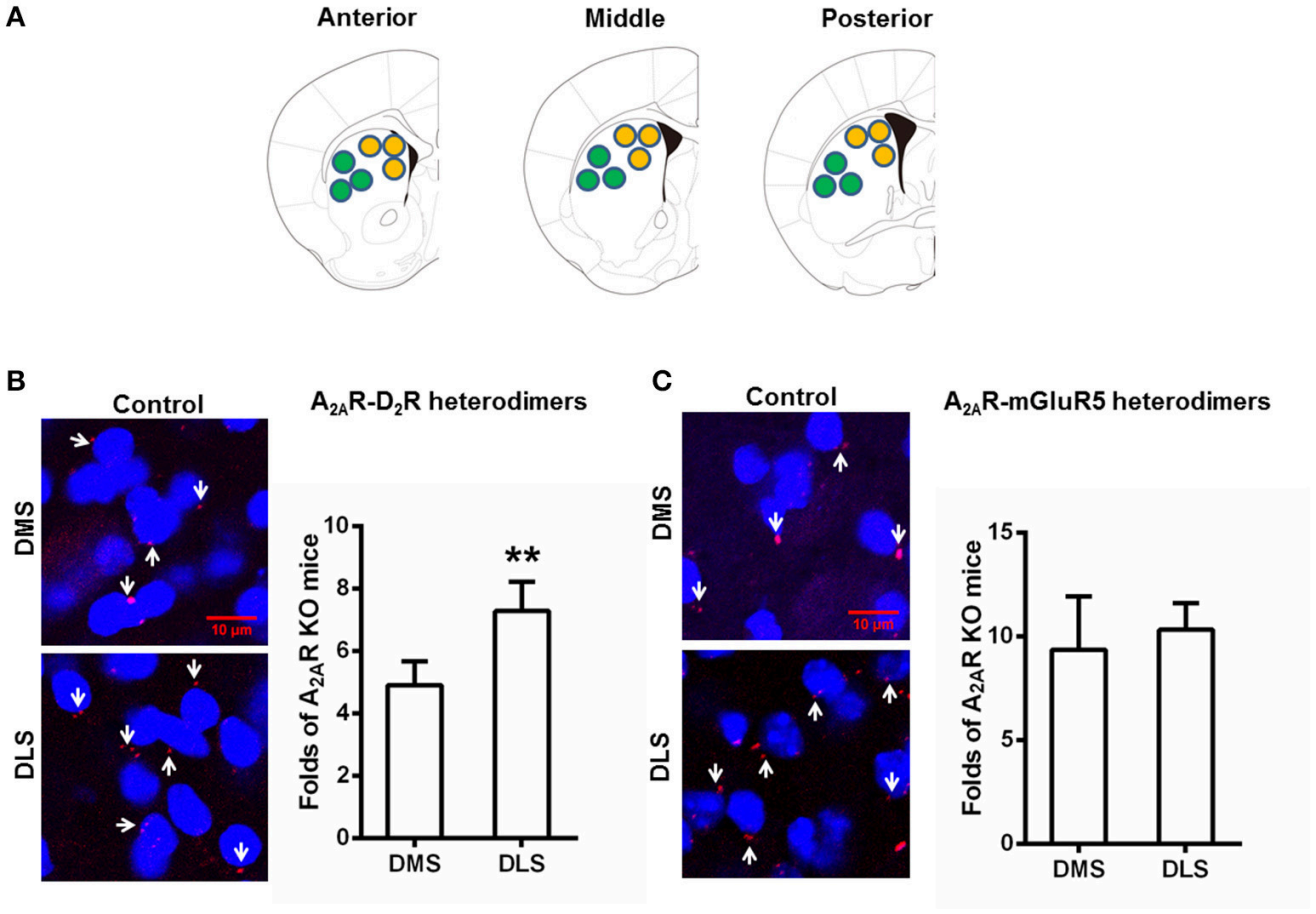
**Heterogeneous distribution of the A_**2A**_R-D_**2**_R heterodimers (but not the A_**2A**_R-mGluR5 heterodimers) between DMS and DLS. (A)** Representative images show: three sections from one brain which was from anterior, middle, and posterior parts of the striatum, respectively. **(B)** Left: representative images show that the A_2A_R-D_2_R heterodimers (as indicated by white arrows) were more abundant in the DLS (lower panels) than DMS (upper panels). Right: quantification of A_2A_R-D_2_R heterodimers confirms that the A_2A_R-D_2_R heterodimers was more abundant in DLS than DMS in the striatum (^**^p = 0.003, paired *t*-test). **(C)** Left: representative images show the A_2A_R-mGluR5 heterodimers (as indicated by white arrows) were indistinctive in both DMS and DLS. Right: quantification of the A_2A_R-mGluR5 heterodimers shows that the A_2A_R-mGluR5 heterodimers displayed no subregional variation between DMS and DLS (*p* = 0.612, paired *t*-test). Data are presented as the mean ± SEM, *n* = 6/group.

After two additional RI60 or RR20 training sessions following devaluation test, mice were sacrificed for PLA detection of the A_2A_R-D_2_R and A_2A_R-mGLuR5 heterodimers in the striatum. We performed PLA analysis according to the procedure described recently (Augusto et al., 2013; Pinna et al., 2014). Three sections from one brain (from anterior, middle, and posterior parts of the striatum, respectively) were rinsed in TBS at room temperature. The sections were incubated with 1% BSA and 0.5% Triton X-100 for 2 h at room temperature for blocking and permeabilization. The mouse anti-A_2A_R (1:300; millipore) and rabbit anti-D_2_R (1:300; millipore)/rabbit anti-mGluR5 (1:300; millipore) were incubated with sections overnight at room temperature. Sections were then rinsed for four times (30 min each time) in TBS with 0.2% Triton X-100 following the manufacturer's protocol. Slices were then incubated at 37°C with the PLA secondary probes (1:5; Olink Bioscience) for 2 h. After rinsing with “Duolink II” Wash Buffer A, the slices were then incubated with the ligation-ligase solution for 30 min at 37°C followed by rinsing with Duolink II Wash Buffer A. Sections were ready for amplification with polymerase (1:40; Olink Bioscience). Then the sections were washed in decreasing concentrations SSC buffers (Olink Bioscience) and mounted on slides. Fluorescent mounting medium (containing DAPI) were applied on the sections. The fluorescence images (three non-overlapping and random microscopic fields respective from DMS and DLS of each brain section) were acquired by confocal microscope (**Figure 2A**). The quantitative analysis was done following the procedure by Bonaventura et al. (2014). The cells surrounded by the red puncta were defined as positive cells (white arrows in Figure 1). The cell number was counted by software “Image J.” Each microscopic field image was quantified as “positive cell number/total cell number.” The quantified value of the experimental mice was normalized to that of A_2A_R KO mice (as the background).

### Immunofluorescence

Mice were deeply anesthetized and then transcardially perfused with 0.01 M PBS (pH = 7.4) followed by ice-cold 4% paraformaldehyde. Brains were post-fixed in 4% paraformaldehyde for 4–6 h at 4°C, and then allowed to equilibrate using gradient sucrose solution (10, 20, and 30%). Immunofluorescence were performed on 30 μm free-floating sections. Free-floating sections were washed in PBS and incubated for 60 min in 0.3% Triton X-100 and 10% normal donkey serum and then incubated with mouse anti-A_2A_R antibody (Millipore, 1:200) and rabbit anti-D_2_R antibody (Millipore, 1:200) at 4°C overnight. Brain sections were incubated with Alexa 488-conjugated secondary antibodies (Invitrogen, 1:1000). The sections were washed and mounted. Fluorescent mounting medium were applied on the sections. Images were acquired by a fluorescence microscope.

### Statistical analysis

Instrumental behavior training and test processes were analyzed using two-way ANOVA for repeated measurements with the training or test sessions as within-subjects effect and the different training procedures as between-subjects effect. In the PLA assay, two-way ANOVA was used with striatal subregions and training procedures as main effects. Paired *t*-test was conducted to compare the distribution difference of A_2A_R-D_2_R and A_2A_R-mGluR5 heterodimers between the DMS and DLS. One-way ANOVA with LSD *post-hoc* was used to compare the distribution variation of A_2A_R-D_2_R heterodimers on the anterior-posterior axes in both DMS and DLS after instrumental learning.

## Result

### Detection of the A_2A_R-D_2_R and A_2A_R-mGluR5 heterodimers by PLA in the striatum

To detect the striatal A_2A_R-D_2_R and A_2A_R-mGluR5 heterodimers by PLA assay, we first confirmed the specificity of the PLA detection of the A_2A_R-D_2_R and A_2A_-mGluR5 heterodimers using the A_2A_R KO and D_2_R KO mice. The specificity of the A_2A_R and D_2_R antibody was evident with highly enriched expression pattern of the A_2A_Rs or D_2_Rs in the striatum by immunofluorescence, which was absent in the A_2A_R or D_2_R KO mice (Figure 1A). Moreover, the specific labeling of the A_2A_R-D_2_R heterodimer signals (cellular membrane) were detected in ~15% of striatal neurons of wild-type mice in close association of DAPI (nuclei; as indicated by white arrow; Figures 1B,C). Importantly, these signals for the A_2A_R-D_2_R protein complexes were essentially absent in the striatum of the A_2A_R KO or D_2_R KO mice (Figures 1B,C), confirming the specificity of the PLA detection of the A_2A_R-D_2_R heterodimers. Similarly, the A_2A_R-mGluR5 heterodimer signals were detected by PLA in the striatum of wild-type mice but not in A_2A_R KO mice, supporting the specificity of PLA detection of the A_2A_R-mGluR5 heterodimers (Figures 1D,E).

### Heterogeneous distribution of the A_2A_R-D_2_R heterodimers (but not the A_2A_R-mGluR5 heterodimers) in the DMS and DLS

Following the confirmation of the specificity of the PLA detection of the A_2A_R-D_2_R and A_2A_R-mGluR5 heterodimers, we examined the heterogeneous distribution of these heterodimers in the DMS and DLS in normal mice. PLA analysis showed that the A_2A_R-D_2_R complexes were more prominent in the DLS than DMS (Figure 2B). Quantitative analysis confirmed the heterogeneous distribution of A_2A_R-D_2_R heterodimers in the striatum (i.e., DLS > DMS; Figure 2B). By contrast, there was no heterogeneous distribution of the A_2A_R-mGluR5 heterodimers in the DMS and DLS by PLA (Figure 2C).

### Random interval schedule promoted habit formation and increased the formation of the striatal A_2A_R-D_2_R heterodimers

**Figure 3:**
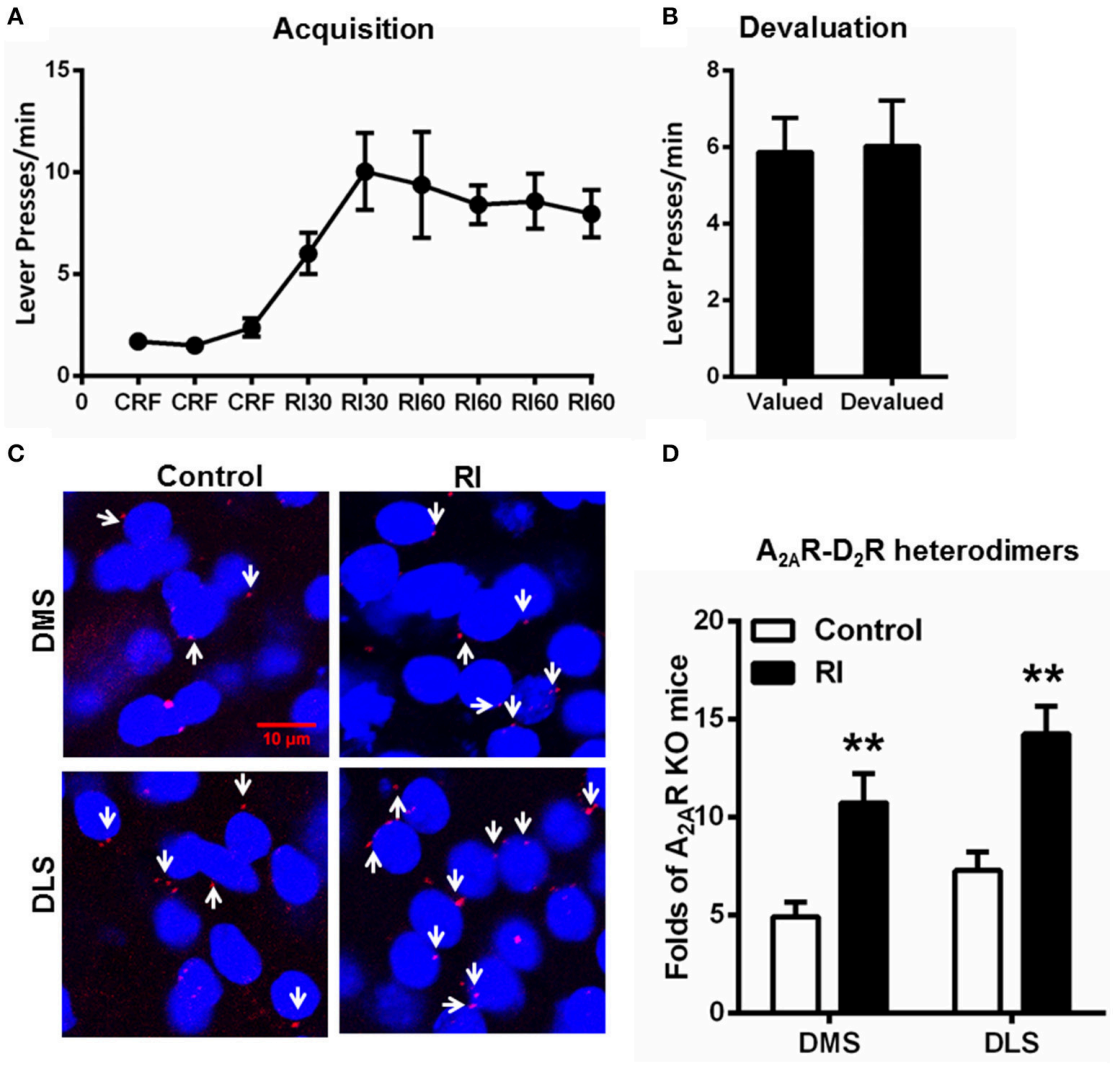
**Random interval schedule promoted habit formation and increased the formation of the striatal A_**2A**_R-D_**2**_R heterodimers. (A)** During the acquisition phase of instrumental behaviors, mice underwent RI training procedure increased their lever presses and reached a plateau at RI60 schedules. **(B)** In the devaluation test, lever presses by mice underwent RI schedule were identical between the valued and devalued conditions, indicating habitual actions (*p* = 0.859, paired *t*-test). **(C)** Representative images show that the A_2A_R-D_2_R heterodimers (as indicated by white arrows) increased markedly after RI training in both DMS and DLS. **(D)** Quantification of A_2A_R-D_2_R heterodimers in DMS and DLS after RI training sessions. The striatal A_2A_R-D_2_R heterodimers in mice underwent RI schedule (forming habit) were significantly increased in both DMS and DLS compared to that of the mice without training (“control”) [DMS: *F*_(1, 10)_ = 12.220, ^**^p = 0.006, DLS: *F*_(1, 10)_ = 16.777, ^**^p = 0.002, two-way ANOVA]. Data are presented as the mean ± SEM from *n* = 6/group.

Following RI training sessions, mice gradually, and steadily increased their lever presses and reached plateau at RI60 schedule (Figure 3A). Consistent with the previous studies (Dickinson et al., 1983; Yin and Knowlton, 2006), devaluation test (Figure 3B) showed that mice trained by RI procedure showed insensitive to outcome devaluation, indicating their habitual action. In association with habit formation after the RI training sessions, we detected the increased formation of the A_2A_R-D_2_R heterodimers compared to mice without training (“control;” Figures 3C,D). Moreover, the A_2A_R-D_2_R heterodimers increased in both DMS and DLS, and accordingly, the heterogeneous pattern of this heterodimers in DMS and DLS persisted after the trainings.

### Random ratio promoted goal-directed behavior without affecting the A_2A_R-D_2_R heterodimer formation in the striatum

**Figure 4:**
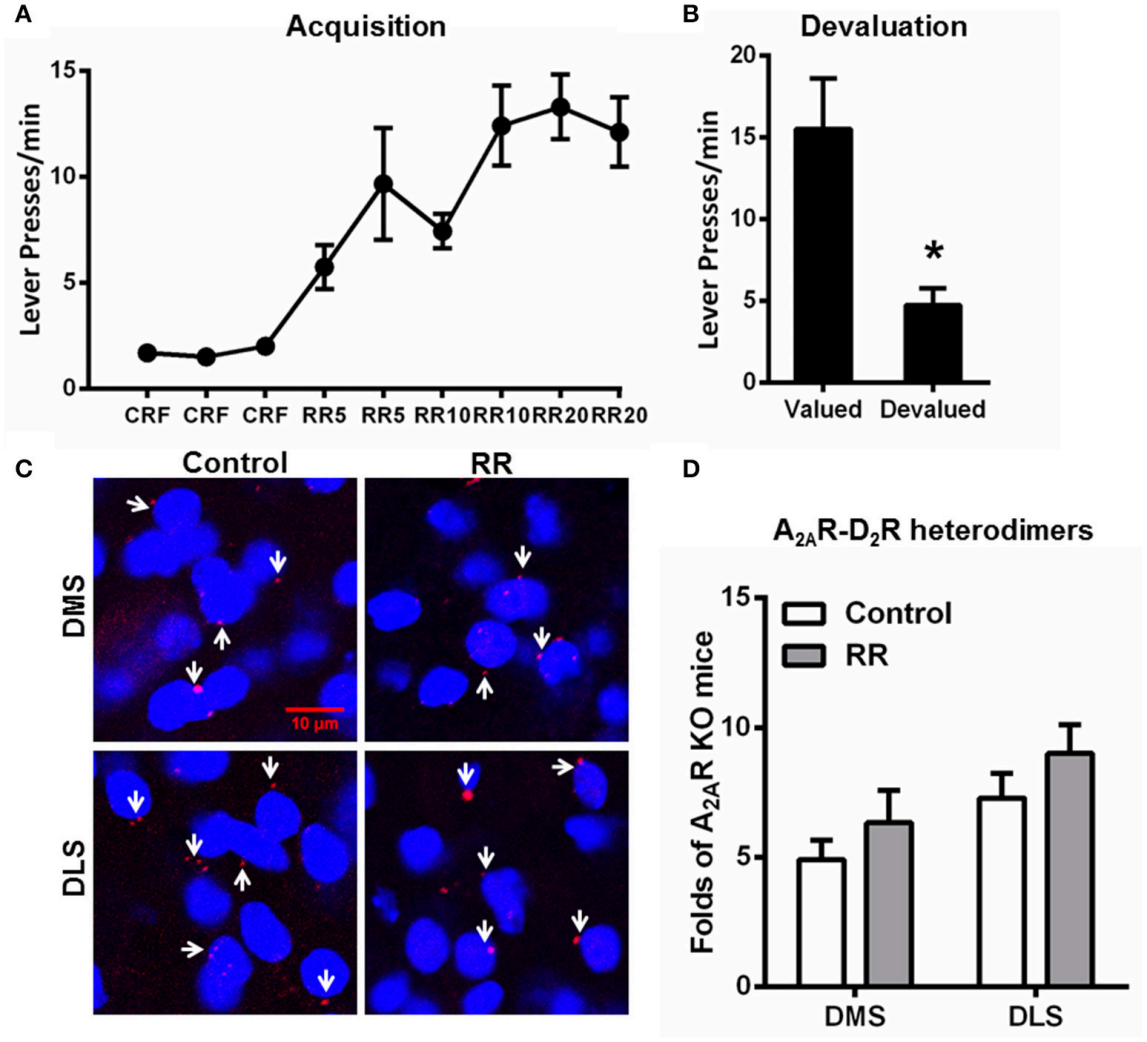
**Random ratio schedule promoted goal-directed behavior without affecting A_**2A**_R-D_**2**_R heterodimers formation in the striatum. (A)** During the acquisition phase of instrumental behaviors, mice underwent RR procedure increased their lever presses, and reached a plateau at RR20 schedules. **(B)** Mice underwent RR schedule showed sensitivity to outcome devaluation by markedly reducing their lever presses, indicating that their behaviors were goal-directed (^*^p = 0.023, paired *t*-test). **(C)** Representative images show the A_2A_R-D_2_R heterodimers by PLA in both DMS and DLS after RR training procedure. **(D)** Quantification of the A_2A_R-D_2_R heterodimers (as indicated by white arrows) by PLA shows that the A_2A_R-D_2_R heterodimers displayed no adaptive changes over RR training schedule [DMS: *F*_(1, 10)_ = 0.926, *p* = 0.359; DLS: *F*_(1, 10)_ = 1.405, *p* = 0.263]. Data are presented as the mean ± SEM from *n* = 6/group.

Over the RR training sessions, mice also gradually and steadily increased their lever presses and reached plateau at RR20 schedule (Figure 4A). Consistent with the previous studies (Dickinson et al., 1983; Yin and Knowlton, 2006), devaluation test showed that mice trained by RR schedule markedly reduced their lever presses in the devalued condition, indicating their goal-directed behavior (Figure 4B). In association with goal-directed behavior after RR training sessions, we did not detect any significant change in the A_2A_R-D_2_R heterodimers compared to mice without training (“control;” Figures 4C,D). Furthermore, the striatal A_2A_R-D_2_R heterodimers underwent RI schedule were significantly increased in both DMS and DLS compared to the RR group [DMS: *F*_(2, 17)_ = 6.351, *p* = 0.010, DLS: *F*_(2, 17)_ = 9.605, *p* = 0.002; ^*^p < 0.05, ^**^p < 0.01; one-way ANOVA with LSD *post-hoc* test, *n* = 6/group]. Thus, the increased association of striatal A_2A_R-D_2_R heterodimers is selectively induced by the RI training schedule in association with habit formation, but not affected by RR training schedule which produced goal-directed behavior.

### A_2A_R-D_2_R heterodimers in the DMS showed prominent increases after RI training on anterior-posterior axes

**Figure 5:**
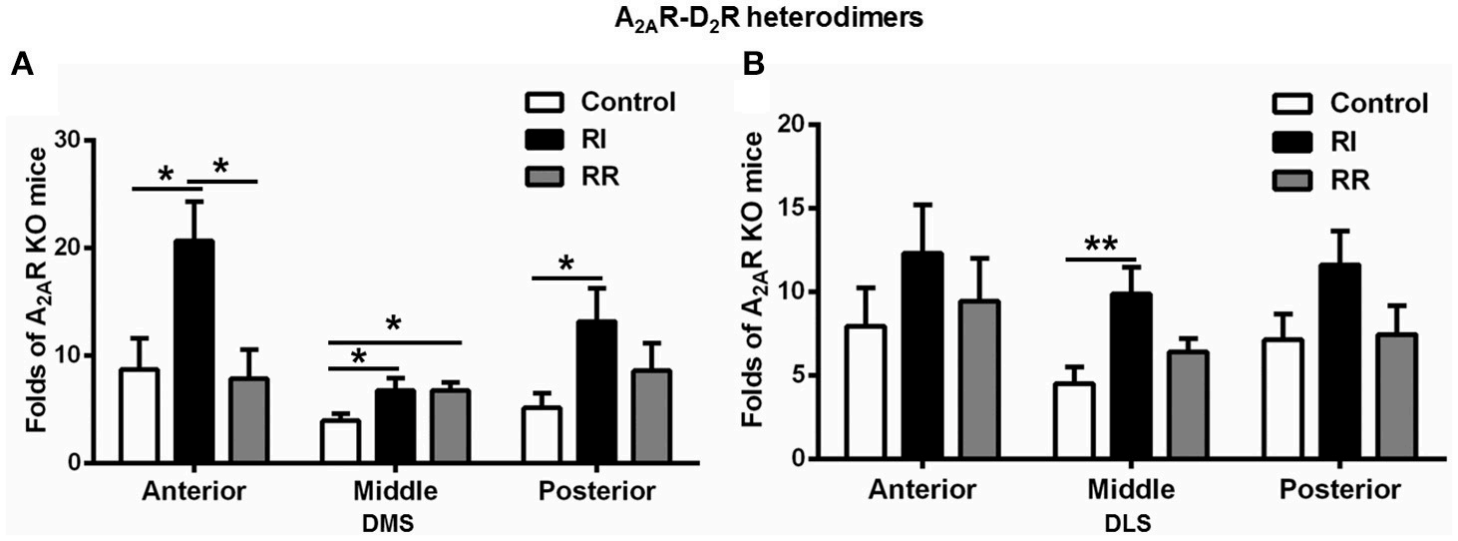
**A_**2A**_R-D_**2**_R heterodimers in the DMS showed more prominent increases after RI training on anterior-posterior axes. (A)** In the DMS, the A_2A_R-D_2_R heterodimers in the anterior and posterior parts were significantly higher in the RI group compared to the control and RR groups [anterior: *F*_(2, 16)_ = 5.135, *p* = 0.021, LSD *post-hoc*: RI vs. control, ^*^p = 0.017, RI vs. RR, ^*^p = 0.011; posterior: *F*_(2, 16)_ = 3.881, ^*^p = 0.046, LSD *post-hoc*: RI vs. control, ^*^p = 0.015]. There was also a relatively small increase in the middle part of the DMS in the RI and RR group compared to the control group [middle: *F*_(2, 16)_ = 3.889, *p* = 0.045, LSD *post-hoc*: RI vs. control, ^*^p = 0.024, RR vs. control, ^*^p = 0.041]. **(B)** In the DLS, the A_2A_R-D_2_R heterodimers in the middle part was higher in the RI group than the control and RR group [*F*_(2, 17)_ = 5.223, ^*^p = 0.019, LSD *post-hoc*: RI vs. control, ^**^p = 0.006]. Data are presented as the mean ± SEM.

We have also performed detailed analysis of the A_2A_R-D_2_R hetrerodimers in three subregions of the DMS and DLS on anterior-posterior axes (i.e., anterior, middle, and posterior, Figure 5). In the DMS, the A_2A_R-D_2_R heterodimers in both the anterior and posterior parts were increased after RI training compared to the control or RR group (Figure 5A). In the DLS, the change in the A_2A_R-D_2_R heterodimers was relatively less pronounced such that the increase was observed only in the middle DLS after RI training compared to the control (Figure 5B). There was no difference in the A_2A_R-D_2_R heterodimers in the anterior, middle and posterior part of the DMS and DLS between the RR or control groups, with exception of apparently a small increase in the middle part of the DMS in the RR group (Figures 5A,B).

### The striatal A_2A_R-D_2_R heterodimers display neither subregional distribution nor adaptive changes after the RI and RR training schedules

**Figure 6:**
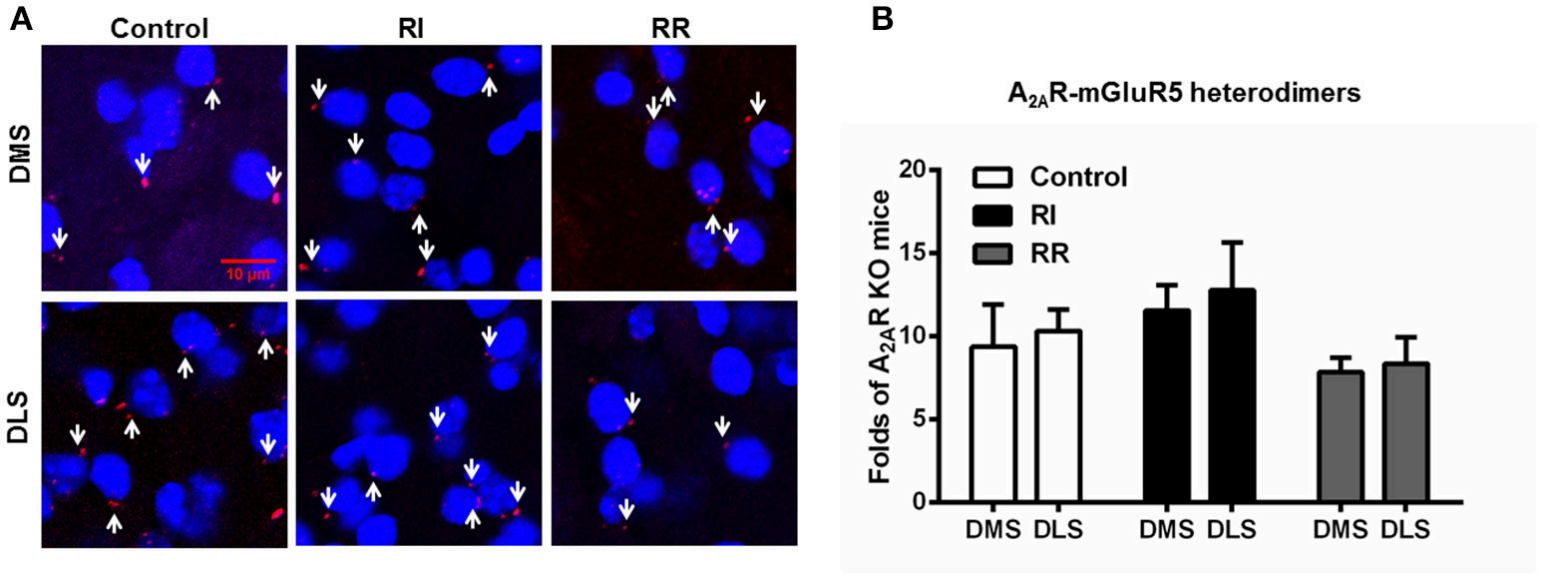
**The striatal A_**2A**_R-mGluR5 heterodimers displayed neither subregional distribution variation nor adaptive changes over the RI and RR training schedules. (A)** Representative images show the A_2A_R-mGluR5 heterodimers by PLA in both DMS and DLS and after different training procedures. **(B)** Quantification of the A_2A_R-mGluR5 heterodimers (as indicated by white arrows) by PLA shows that the A_2A_R-mGluR5 heterodimers displayed neither subregional distribution variation between DMS and DLS nor adaptive changes over RI or RR training schedule [Subregion main effect: *F*_(1, 30)_ = 0.325, *p* = 0.573; Training main effect: *F*_(2, 30)_ = 2.245, *p* = 0.123]. Data are presented as the mean ± SEM from *n* = 6/group.

We also examined the subregional distribution and adaptive changes of the A_2A_R-mGluR5 heterodimers after the RI and RR training schedules (Figure 6). The A_2A_R-mGluR5 heterodimers displayed neither heterogeneous distribution between DMS and DLS nor adaptive changes after the RI training (leading to habit formation) or RR training (leading to goal-directed behavior) sessions (Figures 6A,B). These indicated that such heterogeneous distribution (DLS vs. DMS) and adaptive changes over the instrumental learnings (i.e., increased formation of the heterodimers) were specific for the A_2A_R-D_2_R heterodimers.

## Discussion

The A_2A_R-D_2_R heterodimers have been studied extensively in cultured cells and brain tissues by fluorescence resonance energy transfer (FRET; Torvinen et al., 2005) and receptor binding with biochemical finger printing (Ciruela et al., 2006) and by blocking peptides targeting the presumed A_2A_R-D_2_R interaction site (Azdad et al., 2009) and by co-immunoprecipitation (Ciruela et al., 2006). Since heteromerization of A_2A_Rs and other GPCRs (such as A_2A_R-D_2_R and A_2A_R-mGluR5) has been demonstrated mostly in cultured cell lines with overexpressed recombinant receptors that may result in the creation of many more heterodimers than naturally exist, it is essential to detect the normal distribution of the A_2A_R-D_2_R and A_2A_R-mGluR5 heterodimers in the intact striatum in order to infer their possible physiological functions. However, the direct detection of the A_2A_R-D_2_R and A_2A_R-mGluR5 heterodimers in intact animals and its physiological relevance has been proven to be difficult. Recently, PLA has been developed to detect the presence of the A_2A_R-D_2_R (Trifilieff et al., 2011) and A_2A_R-CD73 heterodimers in the striatum (Augusto et al., 2013). For example, Bonaventura et al. showed that the A_2A_R-D_2_R heterodimers by PLA were reduced in dopamine-depleted caudate-putamen after chronic treatment with L-dopa in non-human primates (Bonaventura et al., 2014). The specificity of the A_2A_R-D_2_R and A_2A_R-mGluR5 heterodimers using PLA was further validated here by demonstrating the detection of the A_2A_R-D_2_R and A_2A_R-mGluR5 heterodimers in the striatum of WT but neither in A_2A_R KO nor in D_2_R KO mice.

Using PLA, we demonstrated the heterogeneous distribution of the A_2A_R-D_2_R heterodimers in DMS and DLS as well as their adaptive changes over the instrumental learning procedures. Given the critical role of the DLS in control of habit formation, the more abundant A_2A_R-D_2_R heterodimers in DLS than DMS under the basal condition may suggest that the A_2A_R-D_2_R heterodimers in DLS may contribute to habit formation. It should be noted that there was no subregional variation in the A_2A_R-mGluR5 heterodimers between the DMS and DLS. Since D_2_R-mediated striatal LTD is preferentially founded in the DLS striatopallidal neurons (Shen et al., 2008), the prominent DLS distribution of A_2A_-D_2_R heterodimers may suggest a possible role of the A_2A_R and D_2_R (rather than the A_2A_R-mGLuR5) interaction in modulating LTD in the DLS.

Importantly, following instrumental learning, our analysis reveals that the formation of the A_2A_R-D_2_R heterodimers increased (by nearly two-folds) over the RI schedule which resulted in habitual behavior compared to their control level. The increased association of the A_2A_R-D_2_R heterodimers was seen selectively after the RI schedule (to promote habit), but was not seen in the mice trained by the RR schedule (to promote goal-directed behavior). Genetic and pharmacological studies have implicated several neurotransmitter and neuromodulator receptors take effects on development of goal-directed and habit behaviors, including D_1_R, D_2_R (Yin et al., 2009), CB_1_R (Hilário et al., 2007), NMDAR (Yin et al., 2005), A_2A_R (Yu et al., 2009; Li et al., 2016), and Gpr6 (Lobo et al., 2007), by alteration of instrumental behaviors. However, to the best of our knowledge, this is the first report on the molecular marker of habitual formation that is selectively induced by the RI (but not the RR) training schedule. This novel molecular correlate of the RI training and possibly habit formation would be useful in molecular dissecting and monitoring habit formation. Moreover, our detailed analysis of A_2A_R-D_2_R heterodimer changes on anterior-posterior axes indicated that the A_2A_R-D_2_R heterodimers in the DMS showed more dynamic changes after RI training in agreement with our recent finding that the DMS A_2A_R signaling plays a major role in control of instrumental learning (Li et al., 2016).

Given the well-documented A_2A_R-D_2_R antagonistic interaction, striatopallidal A_2A_Rs may affect animals' sensitivity to dopamine signaling through the increased A_2A_R-D_2_R heterodimers. Dopamine signaling apparently has more prominent role during the early stage of instrumental learning (goal-directed behavior) than the late stage (habitual behavior; Choi et al., 2005). As the RI training progresses, the formation of the striatal A_2A_R-D_2_R heterodimers increases, resulting in the increased inhibition of the A_2A_R on the D_2_R signaling in the striatopallidal neurons and consequently the less dependence of dopamine signaling at the late stage of instrumental learning. On the other hand, the lack of adaptive changes of the A_2A_R-mGluR5 heterodimers after instrumental learning suggests that striatopallidal A_2A_R activity may preferentially interact with dopamine signaling to modify instrumental learning process.

Since the LTD in striatopallidal neurons, which is modulated by the D_2_R (Kreitzer and Malenka, 2007) and A_2A_R activities (Shen et al., 2008), is associated with a shift in behavioral control from goal-directed action to habitual responding (Nazzaro et al., 2012). We speculated that the increased formation of the A_2A_R-D_2_R heterodimers after the RI learning may increase the inhibition effect of the A_2A_Rs on the D_2_Rs, and consequently reduce D_2_R-mediated striatal LTD in striatopallidal neurons to modify instrumental learning. The prominent changes in the A_2A_R-D_2_R heterodimers in the DMS and its correlation with development of habitual behavior lend support for our interpretation that the increased formation of the A_2A_R-D_2_R heterodimers after RI training augments the inhibitory effect of the A_2A_R on the D_2_R activity and consequently on goal-directed behavior, manifesting as a habitual behavior. Thus, the A_2A_R-D_2_R heterodimers may partially account for recent demonstrations that optogenetic activation of the striatal A_2A_Rs promotes habit (Li et al., 2016) and pharmacological blockade of the A_2A_R promote goal-directed ethanol intake (Nam et al., 2013) and reverse meth-amphetamine-induced facilitation of habitual action (Furlong et al., 2015), albeit the A_2A_R may control habit formation by distinct mechanism other than the A_2A_R-D_2_R heterodimerization. If the functional significance of the A_2A_R-D_2_R heterodimers can be demonstrated by future studies with direct manipulation of these heterodimers (such as the blocking peptide specifically targeting the A_2A_R-D_2_R heterodimers interface; Azdad et al., 2009) in intact animals, the A_2A_R-D_2_R heterodimers may represent a novel therapeutic target for controlling abnormal habit formation associated with obsessive compulsive disorders and relapse of drug addiction.

## Author contributions

YL, YH, ZL, and JC designed the experiment. YL, YH, MC, ZP, FZ, LC, CH, and XC collected the data. YL, YH, MC, YR, XP, ZL, and JC analyzed the data. YL, YH, ZL, and JC wrote the manuscript.

## Funding

This study was sponsored by the National Natural Science Foundation of China grant (No. 81600983), by the Start-up Fund from Wenzhou Medical University (No. 89211010; No. 89212012), the Zhejiang Provincial Special Funds (No. 604161241), the Special Fund for Building National Clinical Key Resource (Key Laboratory of Vision Science, Ministry of Health, No. 601041241), the Central Government Special Fund for Local Universities' Development (No. 474091314) and special BUSM research fund DTD 4-30-14.

### Conflict of interest statement

The authors declare that the research was conducted in the absence of any commercial or financial relationships that could be construed as a potential conflict of interest.
